# The activation of antiviral RNA interference not only exists in neural progenitor cells but also in somatic cells in mammals

**DOI:** 10.1080/22221751.2020.1787798

**Published:** 2020-07-09

**Authors:** Yuqiang Zhang, Zhe Li, Zhi Ye, Yan Xu, Binbin Wang, Congcong Wang, Yunpeng Dai, Jinfeng Lu, Boxun Lu, Wanju Zhang, Yang Li

**Affiliations:** a State Key Laboratory of Genetic Engineering, School of Life Sciences, Fudan University, Shanghai, China; b State Key Laboratory of Medical Neurobiology, School of Life Sciences, Fudan University, Shanghai, China; c Institute for Genomic Medicine, Columbia University Irving Medical Center, New York, NY, USA; d Department of Pathogen Diagnosis and Biosafety, Shanghai Public Health Clinical Center, Fudan University, Shanghai, China

**Keywords:** Zika virus, antiviral immune response, antiviral RNA interference, pathogenesis of ZIKV, immune-compromised mice, virus clearance, mosquito-borne viruses, virus-encoded suppressors of RNA interference

## Abstract

The RNA interference (RNAi) pathway directs an important antiviral immunity mechanism in plants and invertebrates. Recently, we and others have demonstrated that the antiviral RNAi response is also conserved in mammals, at least to five distinct RNA viruses, including Zika virus (ZIKV). ZIKV may preferentially infect neuronal progenitor cells (NPCs) in the developing foetal brain. *Ex vivo* ZIKV infection induces RNAi-mediated antiviral response in human NPCs, but not in the more differentiated NPCs or somatic cells. However, litter is known about the *in vivo* property or function of the virus-derived small-interfering RNAs (vsiRNAs) targeting ZIKV. Here we report a surprising observation: different from *ex vivo* observations, viral small RNAs (vsRNAs) targeting ZIKV were produced *in vivo* upon infection in both central neuron system (CNS) and muscle tissues. In addition, our findings demonstrate the production of canonical vsiRNAs in murine CNS upon antiviral RNAi activation by Sindbis virus (SINV), suggesting the possibility of antiviral immune strategy applied by mammals in the CNS.

## Introduction

The RNA interference (RNAi) pathway directs a conserved antiviral defence mechanism in fungi, plants, insects, nematodes and mammals [1–5]. The antiviral RNAi response has been thoroughly characterized in plants and invertebrates [6]. Genetic and small RNA sequencing studies have shown that antiviral RNAi is initiated by the processing of viral dsRNA precursors synthesized during infection into 21∼23 nt virus-derived small interfering RNAs (siRNAs) by Dicer endonucleases [7]. Subsequently, the viral siRNAs (vsiRNAs) are assembled with an Argonaute protein and other co-factors into RNA-induced silencing complex (RISC) to guide specific virus clearance. Because of the key function for antiviral RNAi, efficient infection of plants and insects with diverse RNA and DNA viruses requires expression of virus-encoded suppressors of RNAi (VSRs), including viral proteins and/or RNA elements, which have been initially identified in transgene RNAi assays [1,7–10].

Recent studies have demonstrated the production of abundant vsiRNAs to target five positive- and negative-strand RNA viruses in mammalian cells, including influenza A virus (IAV), human enterovirus 71 (HEV71) and Zika virus (ZIKV) [11–15]. ZIKV is a flavivirus in the *Flaviviridae* family transmitted sexually, vertically and by mosquitoes [16]. ZIKV preferentially infects neuronal progenitor cells (NPCs) in the developing foetal brain and has also been found in tissues outside the central nervous system (CNS), including the eyes, testis, and female reproductive tract organs [16–19]. However, few reports focus on the basis of ZIKV cellular and tissue tropism [20–22]. ZIKV infection induces a series of host cell innate immune responses, pro-inflammatory responses, and humoral immune responses by producing protective and neutralizing antibodies in humans [17,23,24]. Qin and colleagues recently revealed that *ex vivo* ZIKV infection induces RNAi-mediated antiviral response in human NPCs (hNPCs), but not in neurons differentiated from them [11,25]. An earlier study also reported that canonical vsiRNAs were detected in mouse embryonic stem cells (mESCs) by infection with the picornavirus encephalomyocarditis virus (EMCV) and production of these vsiRNAs by mESCs was greatly reduced upon cell differentiation [13]. These two studies suggest that Dicer-mediated processing of dsRNA replication intermediates into vsiRNAs may occur in stem cells, but not or less efficiently in differentiated cells by *ex vivo* infections [2,11,13,26].

Meanwhile, whether this is the case *in vivo* is completely unknown. This is critical because ZIKV pathogenesis has been of extreme global public health interest since its outbreak in 2015, and a better understanding of interactions with the host would provide insight into molecular mechanisms driving the severe neurological outcomes of ZIKV disease [17]. Small animal models are useful for the evaluation of antiviral agents and vaccines during preclinical studies. The threats posed by ZIKV have prompted the development of various *in vivo* animal models to better understand the pathogenesis of ZIKV, and these models include either immune-compromised or immunocompetent neonatal and adult mice [16,27–29]. Here we showed that the *in vivo* characterization of mouse viral siRNAs produced by virus infections, providing undiscovered features of antiviral RNAi response in mammals.

## Materials and methods

### Cell culture

Baby hamster kidney cells (BHK) and African green monkey kidney epithelial cells (Vero) were purchased from the American Type Culture Collection (ATCC). Dicer knockout 293 T cell line (4-25 cell line) was a gift from B. Cullen. Both cell lines were cultured in Dulbecco's modified Eagle's medium (DMEM, Gibco) containing 10% foetal bovine serum (Gibco).

### Viruses

IAV, PR8/delNS1 (NS1 deletion mutant) was a gift from A. Garcıa-Sastre and P. Palese. ZIKV strain (SZ01) was provided from Shanghai Public Health Clinical Center, Fudan University. ZIKV stocks were propagated in Vero cells after inoculating at a multiplicity of infection (MOI) of 0.01 and harvesting supernatants after 5 days. Sindbis virus (SINV) were rescued from the plasmid of pSVN1, which was gift from Dr. C.M. Rice. Briefly, the plasmids were linearized with *Xho*I and then SINV genomic RNAs were transcribed *in vitro* using an SP6 mMESSAGE mMACHINE kit (Ambion). Purified SINV genomic RNAs were transfected into BHK cells by TransIT®-mRNA Transfection Kit (Mirus Bio, WI). Viruses were harvested and tittered as previously described [30]. PR8/delNS1, ZIKV, SINV titre were 5 × 10^5^, 2 × 10^6^, 1 × 10^7^ plaque-forming units (PFU)/mL respectively.

### Animals

BALB/c and C57BL/6 mice were purchased from Shanghai SLAC Laboratory Animal Co., Ltd. *Ifnar1^−/–^* mice were bought from Cyagen Biosciences (Suzhou, China). All the animal experiments in China were carried out under the guidelines of the Institutional Animal Care and Use Committee, Fudan University.

### ZIKV infection

Vero cells were infected with ZIKV at 0.1 MOI. At 5 days post-infection (dpi), infected cells were harvested for the extraction of total protein and RNA using TRIzol (Invitrogen, Carlsbad, CA) according to the manufacturer's protocol. 1 mL TRIzol reagent add into 50 mg of the brain and hindlimb tissue and homogenized using homogenizer. The lysate centrifuge for 5 min at 12,000g at low temperature and a clear supernatant was transferred to a new tube. Then, total RNA was extracted by isopropanol methods as described [31].

BALB/c, C57BL/6 and *Ifnar1^−/–^* suckling mice were inoculated with ZIKV of 10^4^ PFU by intraperitoneal injection (i.p.). Total RNAs were extracted from the brain or hindlimb muscle tissues of BALB/c, C57BL/6 and *Ifnar1^−/−^* suckling mice at 8, 5 and 4 dpi, respectively. Virus accumulation of the mice tissues were determined by RT-qPCR and Western blotting. Primers were shown in Table S1.

### Co-immunoprecipitation (Co-IP)

*Ifnar1^−/−^* suckling mice infection with ZIKV or the same volume of DMEM (mock) by anti-pan Argonaute (Ago) antibody (Millipore, Billerica, MA; catalogue number MABE56) or mouse IgG antibodies (Santa Cruz Biotechnology, Santa Cruz, CA; catalogue number sc-2027) were essential as described. Briefly, 100 μg of muscle tissue lysates in 1 ml RIPA were pre-cleared by sequential incubation with 3 μg of rabbit or mouse IgG and 15 μl of protein A/G PLUS-Agarose beads (Santa Cruz Biotechnology, Santa Cruz, CA; catalogue number sc-2003). 3 μg of anti-pan Ago antibodies or mouse IgG antibodies immobilized to protein A/G PLUS-Agarose beads were then incubated with the pre-cleared cell lysates for 2 h at 4 °C. After extensive washes, the precipitated complexes were used for RNA extraction by TRIzol and the total RNAs obtained were used for the construction of small RNA libraries as described.

### Western blotting analyses

Western blotting analysis was performed as described previously [15]. Antibodies to IAV-NS1 was described previously [15]. Antibodies to human Dicer (hDicer) (Santa Cruz Biotechnology; catalogue number sc136979), β-actin (Cell Signaling Technology; catalogue number 4967L) and ZIKV capsid (GeneTex; catalogue number GTX133317) were sourced from commercial suppliers.

### SINV viruses production

SINV_GFP_, SINV_mC_, SINV_B2,_ SINV_NS1_, SINV_NS4A_, SINV_NS4B_ and SINV_capsid_ viruses were rescued separately from the plasmids of pTE/5′2J/GFP, pTE/5′2J/mC, pTE/5′2J/B2, pTE/5′2J/NS1, pTE/5′2J/NS4A, pTE/5′2J/NS4B and pTE/5′2J/capsid. The pTE/5′2J/GFP (SINV expression EGFP) and pTE/5′2J were gifts from Dr C.M. Rice. In pTE/5′2J, the gene of interest can be inserted at a multiple cloning site (MCS) downstream of the duplicated subgenomic promotor sequence. These plasmids were constructed by ligating PCR products of ZIKV genome RNA (1-376 nt), NoV-B2, IAV-NS1 and ZIKV-NS4A, NS4B, Capsid flanked by *Xba*I sites at 5′ and *Apa*I sites at 3′ into the MCS of pTE/5′2J. Briefly, these plasmids were linearized with *Xho*I and then SINV genomic RNAs were transcribed *in vitro* using an SP6 mMESSAGE mMACHINE kit (Ambion). Purified SINV genomic RNAs were transfected into BHK cells by TransIT®-mRNA Transfection Kit (Mirus Bio, WI). Viruses were harvested and titred as previously described [30]. SINV_GFP_, SINV_mC_, SINV_B2,_ SINV_NS1_, SINV_capsid_, SINV_NS4A_ and SINV_NS4B_ viruses titre were 5×10^6^, 2×10^6^, 1×10^6^, 1.2×10^7^, 1×10^7^, 2×10^6^, 1×10^6^ PFU/mL, respectively.

### In vivo recombinant SINV reporter experiments

For *Ifnar1^−/−^* suckling mice, seven-day-old mice were inoculated by i.p. with ZIKV (10^4^ PFU) or with the same volume of DMEM (mock). Two days after inoculation, the mice were infected by i.p. with SINV_GFP_ or SINV_mC_ viruses of 500 PFU. Each group of four suckling mice were euthanized one day after SINV infection to determine virus titres in the hindlimb tissue by RT-qPCR. Primers were shown in Table S1.

### Cell culture transfection and infection

To determine the activity of VSRs, hDcr-KO 293 T cells seeded in a 6 cm dish at a density of 2.5 × 10^6^ per dish were co-transfected with 8 µg of the hDicer expression plasmid (Addgene no.19873) with mock or one (4 µg) of the following plasmids, pcDNA-IAV-NS1 and pcDNA-ZIKV-Capsid. At 6 h after co-transfection, the hDcr-KO 293 T cells were infected by PR8/delNS1 (MOI = 1), and the infected cells were collected for the extraction of total protein and RNA using TRIzol at 24 h after infection.

### SINV infection

BHK cells were infected with SINV (MOI=0.01) and harvested for the extraction of total RNAs after 24 h infection. 100 PFU of SINV was inoculated to 6–8 days old BALB/c suckling mice by i.p. and the total RNAs were extracted from the brain tissue of mice after 3 dpi. BALB/c or C57BL/6 adult mice were challenged by left lateral cerebral ventricle injection using a brain solid positioner (KD Scientific, LEGATO130) with 5×10^2^ PFU of SINV. Total RNAs were extracted from the total hindlimb, brain tissue or brainstem, cerebellum, striatum, hippocampus and cerebral cortex of mice after 3 dpi, respectively.

### Stem-loop RT-qPCR

RNA samples were reverse transcribed to cDNA using either random hexamers for detecting actin or gene-specific stem-loop RT primers. 1 µg total RNA was incubated with 5x gRNA wiper Mix (Vazyme; catalogue number R312) and incubated for 2 min at 42°C. The reactions were cooled to room temperature and RT Mix and oligo (Vazyme; catalogue number R312) were added. The reactions were incubated in an Bio-rad T100 Thermocycler for 5 min at 25°C, 15 min at 50°C, 5 min at 85°C and held at 4°C according to instruction. The reactions were cooled to room temperature and RT-qPCR was performed using a standard universial SYBR qPCR kit (Vazyme; catalogue number Q711) on Bio-Rad CFX96 system. All primers used were listed in Table S2.

### Construction of small RNA libraries

RNA preparations in this study were used for the construction of small RNA libraries by the method that depends on the 5′ monophosphate of small RNAs as described previously with the TruSeq Small RNA Sample Preparation Kit of Illumina (San Diego, CA) [14].

### Deep sequencing and bioinformatic analysis of small RNAs

Libraries of small RNAs were cloned from the RNA samples (mice n=3, cell samples, repeat once) and sequenced by Illumina HiSeq 2000/2500. 18 libraries in total were sequenced from this work (Table S3). Small RNA reads were removed from adapter sequences, and were mapped to the virus genome references or compared to mature miRNAs. Mapping was done by Bowtie 1.1.2 with a perfect match. All of the references used were downloaded from web sources. Subsequent bioinformatics analysis of virus-derived small RNAs was carried out using in-house Perl scripts as described previously [15]. Pairs of complementary 22-nt vsiRNAs in each library with different base-pairing lengths were computed using a previously described algorithm [15]. The reference sequences used in this study are either identical with those described previously or as listed below:

1. ZIKV: KX253996.1
2. SINV: j02363.1
3. PR8/delNS1: Obtained from A/Puerto Rico/8/34 (H1N1) (PR8-WT) by deleting nucleotides 57–528 in the NS segment. The sequence of PR8-WT was downloaded from NCBI: AF389115.1, AF389116.1, AF389117.1, AF389118.1, AF389119.1, AF389120.1, AF389121.1 and AF389122.1.
4. Mature miRNAs: miRBase 21 (http://www.mirbase.org/).

## Results

### The properties of viral small RNAs produced in Vero cells and neonatal mice with ZIKV infection

**Figure 1.**
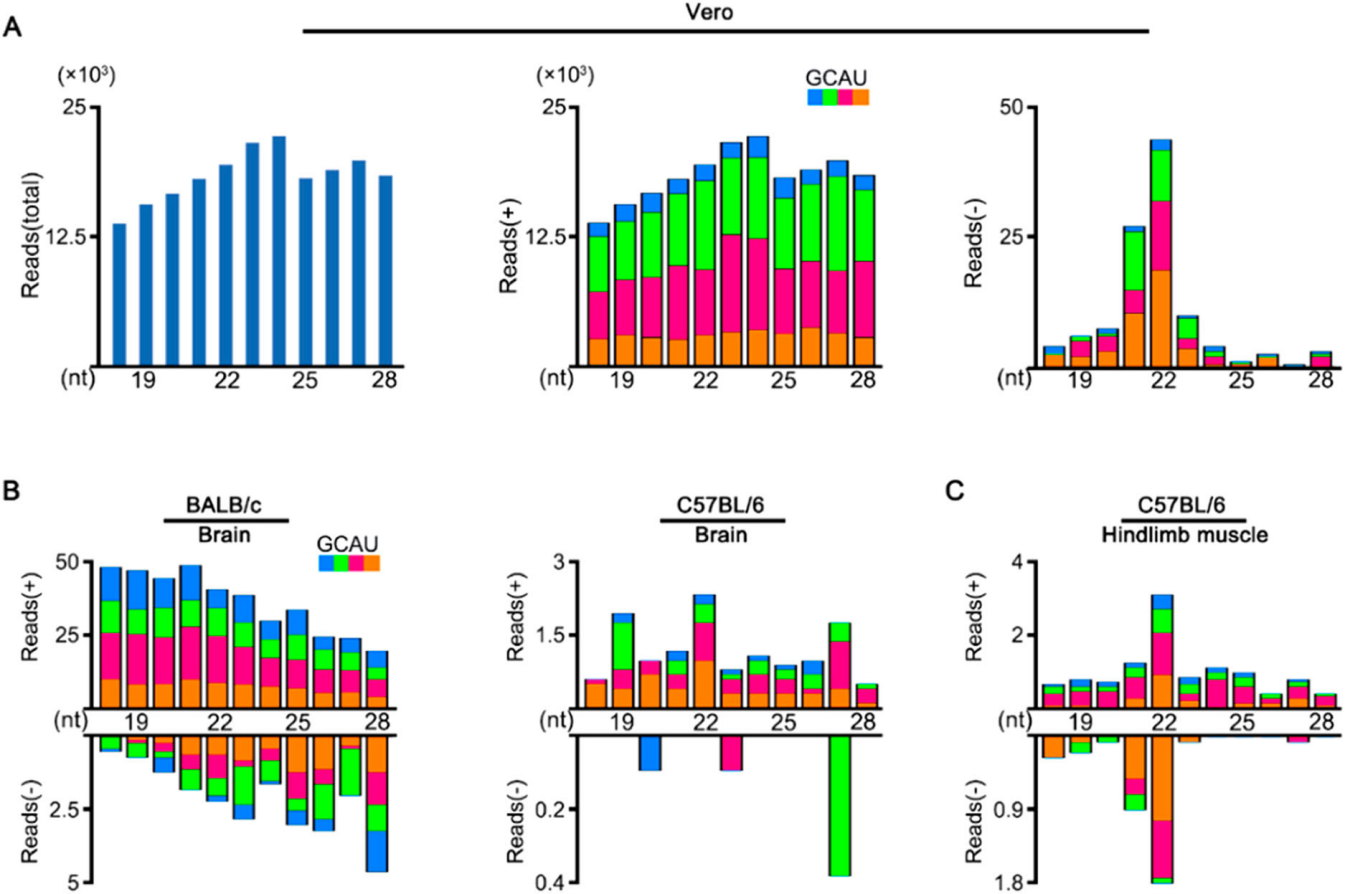
Production of vsRNAs in Vero cells and WT mice infected with ZIKV. (A) Size distribution of 18- to 28-nt ZIKV-derived small RNAs reads sequenced from Vero cells of both strands (left), of only genomic strand (middle), or of only antigenomic strand (right). Reads are shown as per million mature miRNAs. Reads (+) represents vsRNAs derived from viral genome, reads (-) represents vsRNAs derived from viral anti-genome. 5′ terminal nucleotide of vsRNAs is indicated by colour. (B, C) Size distribution of 18- to 28-nt ZIKV-derived small RNAs reads sequenced from brain tissue (B) and hindlimb muscle tissue (C) of BALB/c or C57BL/6 suckling mice infected with ZIKV.

To investigate the vsRNAs properties, we first analysed the small RNA library constructed from African green monkey kidney (Vero) cells infected with ZIKV (Figure 1(A)). Although the viral small RNAs derived from ZIKV genome strand were highly abundant, we detected no significant peak at 21–23 nucleotides (nt) (Figure 1(A, middle)). In contrast, vsRNAs from ZIKV antigenome strand exhibited a canonical size distribution enriched at 22 nt, despite of their relatively low abundance compared to genomic vsRNAs (Figure 1(A, right)). We next inoculated the BALB/c and C57BL/6 suckling mice with ZIKV by i.p., as previous studies indicate weaned wild-type (WT) mice were not susceptible to ZIKV infection [16,27]. These suckling mice showed only weak symptoms and low accumulations of viral RNAs in tested tissues after injection (Figure S1). The profile of ZIKV-derived small RNAs sequenced from the brain of these suckling mice exhibited no size preference expected for Dicer products. (Figure 1(B)). Interestingly, the vsRNAs sequenced from hindlimb muscle tissue of the C57BL/6 suckling mice showed a 22 nt dominant size distribution for both viral genome and antigenome strands (Figure 1(C)), suggesting the generation of vsiRNAs in muscle tissue.

### The abundant vsiRNAs produced in Ifnar1^−/−^ mouse with ZIKV infection

**Figure 2.**
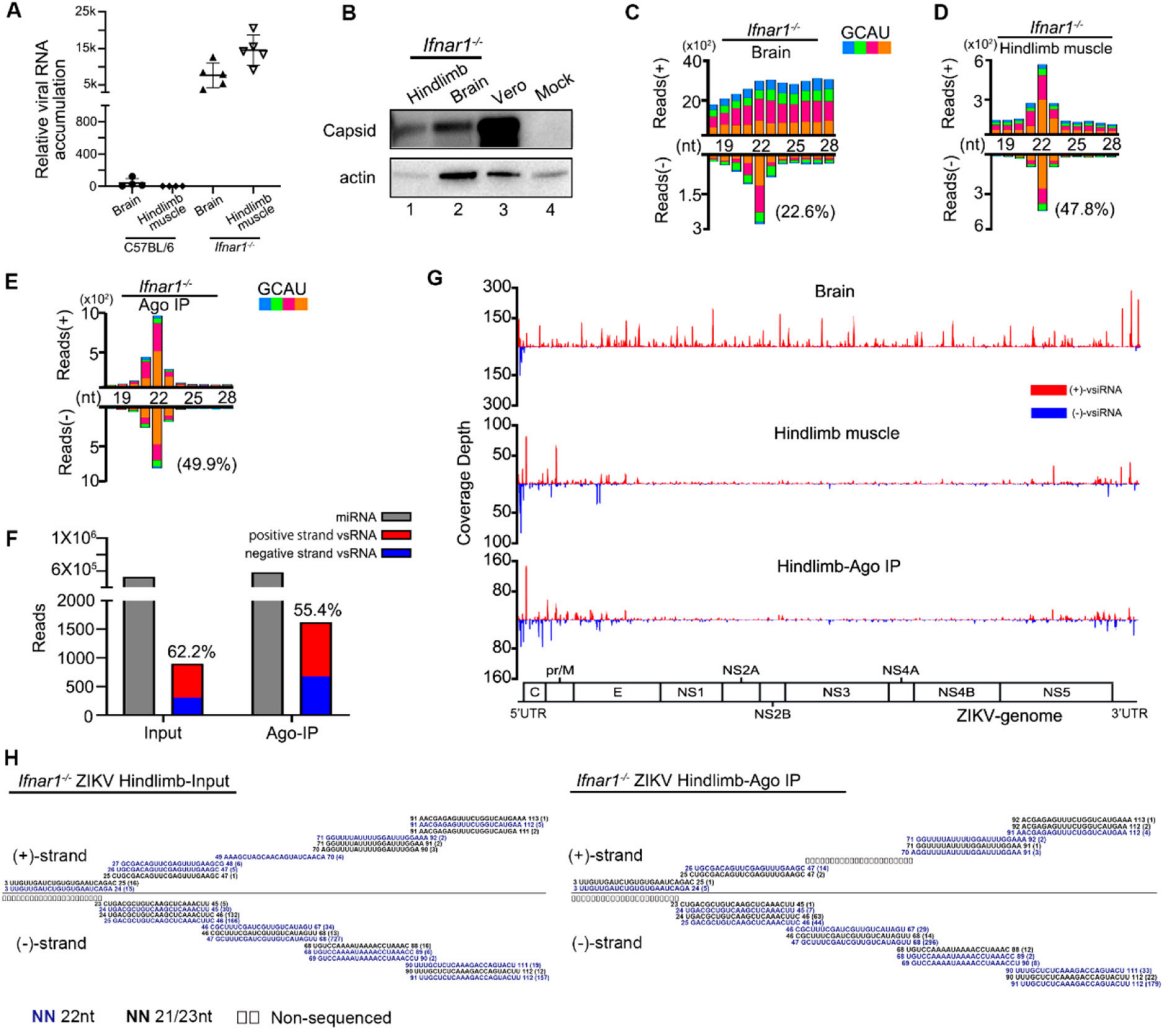
Profile of vsiRNAs in *Ifnar1^−/−^* suckling mice infected with ZIKV. (A) The relative viral RNA accumulation determined by RT-qPCR from brain and hindlimb muscle of C57BL/6 and *Ifnar1^−/−^* suckling mice infected with ZIKV (n=4∼5 per group). (B) Expression levels of ZIKV capsid protein measured in hindlimb muscle and brain of *Ifnar1^−/−^* suckling mice and in Vero cells. Staining of β-actin was used as a loading control. Mock indicated the muscle tissue of naïve *Ifnar1^−/−^* suckling mice. (C-E) Size distribution of vsiRNAs sequenced from brain (C) and hindlimb muscle (D, E) of *Ifnar1^−/−^* suckling mice infected with ZIKV, either without (C, D) or with (E) co-immunoprecipitation by antibodies specific to AGOs (Ago-IP). Reads are shown as per million mature miRNAs. 5′ terminal nucleotide of vsRNAs is indicated by colour. 1U% of 21- to 23-nt vsiRNAs in each library is shown in parentheses. (F) Counts (per million 21- to 23-nt total reads) of mature miRNAs and ZIKV 21-23nt vsiRNAs from the libraries of Input (D) and Ago-IP (E). Red represents positive strand vsRNA, blue represents negative strand vsRNA. (G) Genomic coverage depth by 21- to 23-nt vsiRNAs sequenced from brain (top), hindlimb muscle (middle), and Ago-IP (bottom) from *Ifnar1^−/−^* suckling mice infected with ZIKV at 4 dpi. (H) Read sequences along the 100 nt segment at the 5′-terminal of ZIKV genome from hindlimb muscle (left) and co-immunoprecipitation by antibodies specific to AGOs (right) of *Ifnar1^−/−^* suckling mice infected with ZIKV at 4 dpi. Read counts (in brackets), read lengths, genomic positions are indicated.

The positive result in the muscle tissue of WT suckling mice encouraged us to increase ZIKV infection efficiency for a better *in vivo* characterization of ZIKV vsiRNAs. ZIKV caused disease in the *Ifnar1^−/−^* mouse (which cannot respond to IFN-α/β) with high replication level and spread to many tissues including the CNS, highlighting the utility of the *Ifnar1^−/−^* mouse model for studies of ZIKV pathogenesis [16,27,28]. Therefore, we next inoculated the *Ifnar1*^−/−^ suckling mice with ZIKV by i.p.. As expected, the *in vivo* accumulation of ZIKV in *Ifnar1*^−/−^ mice was significantly enhanced compared to WT C57BL/6 mice (Figure 2(A,B) and Figure S1(B)). Unlike the observed no defined peak at a specific length of both genome and antigenome stands of vsRNA from the brain tissue of infected WT mice (Figure 1(B)), the ZIKV antigenome vsRNAs from the brain tissue of *Ifnar1*^−/−^ mice exhibited the 22-nt peak for size distribution, while the genome vsRNAs were still distributed similarly to the result detected in Vero cells (Figure 2(C), Figure S2(A) and S3(D)). Compared with the brain tissue, the ZIKV vsRNAs from the hindlimb muscle tissue exhibited the “hallmark” of vsiRNAs, with 66% of the vsRNA reads enriched within 21- to 23- nt size range, a dominant population at 22-nt for both genome and antigenome strands and the 20-nt perfectly base-paired duplexes with 2-nt 3′ overhang. (Figure 2(D), Figure S2(B), S3(E) and Table S3). The abundance (normalized by total reads) and size distribution of ZIKV vsiRNAs from the muscle tissue of *Ifnar1*^−/−^ suckling mice are similar to that reported in hNPCs (Figure S3(B)) [11]. Moreover, we performed stem-loop RT-qPCR to detect some ZIKV-derived vsiRNAs and murine miRNAs. Our results showed that these vsiRNAs were detectable at 2 dpi, and accumulated at a higher level at 4 dpi (Figure S4). To further investigate whether the ZIKV vsiRNAs are loaded into RISCs, we next sequenced the small RNAs in the immunoprecipitants by a pan-Argonaute antibody to pull-down mouse Argonaute proteins and their associated RNAs obtained from the muscle of ZIKV infected *Ifnar1*^−/−^ infant mice (Figure 2(E), Figure S2(C) and S3(F)). The abundance of Argonaute-bound vsiRNAs was highly enriched compared to that of total vsiRNAs without immunoprecipitation (Figure 2(F)). Similar to the size pattern of total vsiRNAs, 90% of the Argonaute-bound vsiRNAs were within the size range of 21- to 23- nt with a dominant 22-nt peak, and were approximately equally derived from genome and antigenome strands (Figure 2(E,F), and Table S3). Furthermore, we detected similar viral genome distribution patterns of ZIKV vsiRNAs between the total and Argonaute-bound populations, both with vsiRNA hot spots at the terminal regions of the viral RNA genome (Figure 2(G,H)).

### The vsiRNAs of ZIKV confer specific antiviral activity in vivo

**Figure 3.**
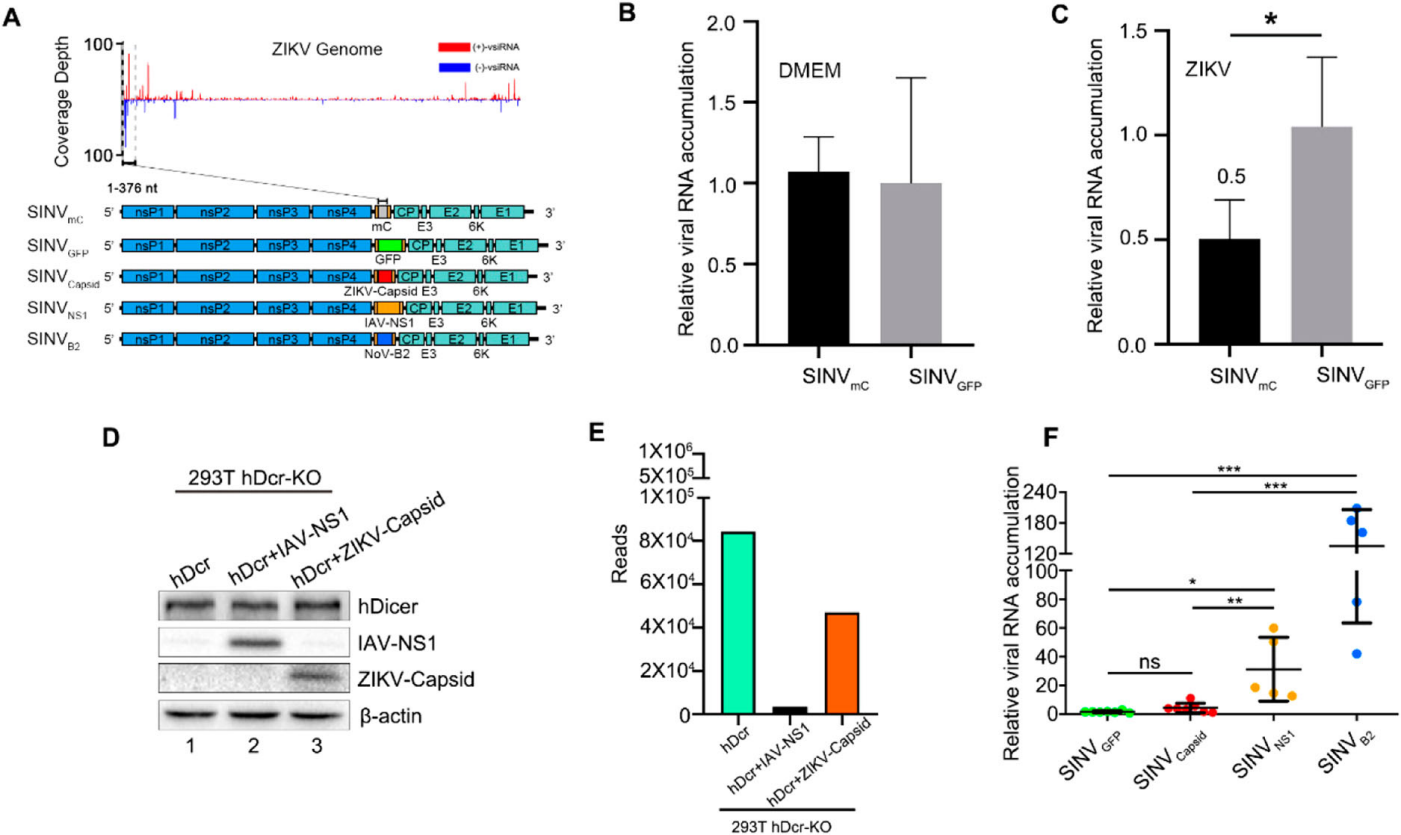
The specific viral siRNAs of ZIKV induce homology-dependent virus resistance. (A) Diagram of recombinant SINV_mC_, SINV_GFP_, SINV_Capsid_, SINV_NS1_, SINV_B2_. (B, C) Relative viral accumulation levels in *Ifnar1^−/−^* suckling mice first challenged with culture medium DMEM (B) or ZIKV (C), and then inoculated by SINV_mC_ and SINV_GFP_ respectively. The SINV-nsP2 mRNA levels were measured at 1 dpi (n=4 per group). * indicates *p*<0.05, Student's t-test. (D) Suppression of inﬂuenza vsiRNA biogenesis in PR8/delNS1-infected hDcr-KO 293 T cells ectopically expressing hDcr, hDcr + IAV-NS1 and hDcr + ZIKV-Capsid, protein expression levels determined by western blotting. Staining of β-actin was used as a loading control. (E) Counts (per million 18- to 28-nt total reads) of IAV-derived siRNAs sequenced from the cells in (D) infected by PR8/delNS1. (F) The relative viral accumulation levels determined by RT-qPCR from hindlimb muscle of BALB/c suckling mice infected with SINV_GFP_, SINV_Capsid_, SINV_NS1_ or SINV_B2_ or at 3 dpi (n=5∼7 per group). ns indicates no significant, * indicates *p*<0.05, ** indicates *p*<0.01, *** indicates *p*<0.001, Student's t-test.

To determine whether the ZIKV infection trigger the homology-dependent viral RNA degradation guided by the vsiRNAs, we constructed a recombinant SINV engineered to contain an insert from 5′ end of ZIKV genome (SINV_mC_), which we found to be targeted by the high density of vsiRNAs in ZIKV infected mice (Figure 3(A)) [30]. SINV_mC_ replicated to significantly lower levels compared to SINV_GFP_ in the *Ifnar1*^−/−^ suckling mice pre-inoculated with ZIKV, but not in mice pre-inoculated with DMEM, suggesting ZIKV vsiRNAs were able to guide the homology-dependent antiviral RNAi (Figure 3(B,C)). We next investigated whether ZIKV encodes VSR to suppress the antiviral RNAi. Recent studies show that ZIKV capsid has a potential interaction with Dicer and would be a candidate of VSR [32,33]. To test this hypothesis, we investigated the ZIKV capsid protein in our VSRs assay system, compared with an identified VSR by an authentic virus infection of human somatic cells (Figure 3(D)) [15]. Our results showed that the production of the vsiRNAs of deficient influenza A virus (IAV) was significantly suppressed by its NS1 protein, a known viral suppressor of RNAi, but not by ZIKV capsid protein (Figure 3(E) and Figure S5(A))[34,35]. To further investigate the role of ZIKV capsid protein in modulating alphavirus replication *in vivo*, suckling mice were infected with equivalent titres of SINV_capsid_, SINV_NS1_, SINV_B2_ (B2 protein is a known viral suppressor of RNAi from Nodamura virus, (NoV)) and SINV_GFP_, which carrying double subgenomic SINV derived from the infectious cDNA clone pTE/5′2J (Figure 3(A)) [3,30,36,37]. The viral RNA of SINV_NS1_ and SINV_B2_ accumulated to significantly higher levels in the presence of functional IAV NS1 and NoV B2 proteins compared to SINV_GFP_, but such enhancement was undetectable for SINV_capsid_ (Figure 3(F)). Moreover, we tested other potential VSRs, NS4A and NS4B, proteins of ZIKV, and none of them could enhance the replication of recombinant SINV *in vivo* (Figure S5B) [17,32]. These results suggested a possible absence of strong viral suppression of vsiRNA biogenesis upon ZIKV infection and provided an explanation regarding why we could detect abundant vsiRNAs in the mice infected by even WT ZIKV.

### Characterization of SINV-derived vsRNAs in BHK cells and murine CNS

Although immune-compromised mouse strains are permissive for ZIKV infection and displayed various neurologic signs of disease, WT mice generally display only a very transient viral infection after ZIKV challenge [16,27]. To better understand the contributions of the vsiRNA-mediated antiviral mechanism of neurological disease-related viral infection, we applied SINV in our *in vivo* study to inoculate WT mice. SINV is widely used as a model system for studying the pathogenesis of virus-induced neurological disease [38–42]. It is a mosquito-borne prototype alphavirus in the *Togaviridae* family with an enveloped plus-stranded RNA genome, primarily targets neurons for its infection in the CNS, and causes encephalomyelitis in mice [39].

**Figure 4.**
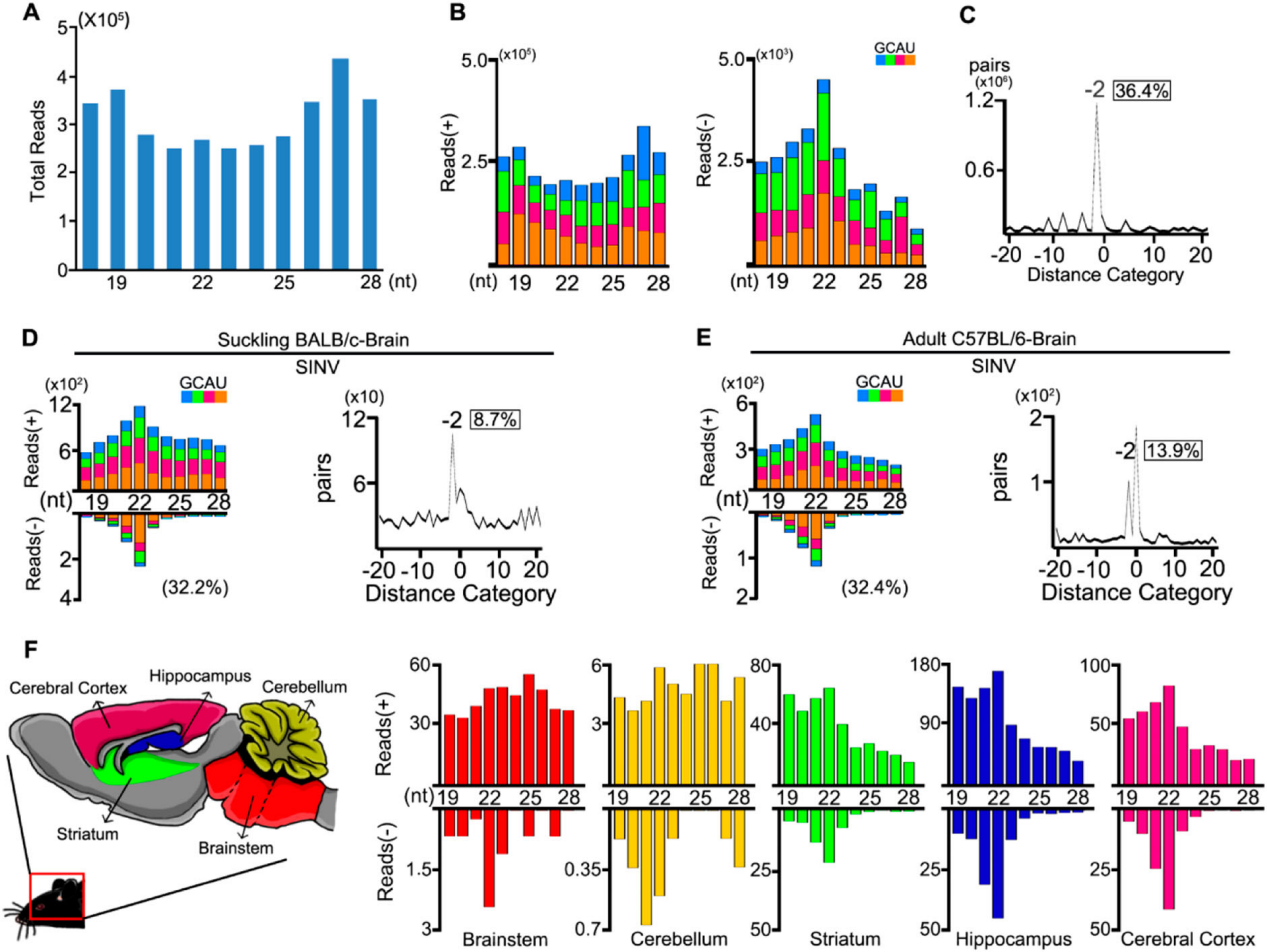
Production of vsRNAs in BHK cells and brain tissues of WT mice infected with SINV. (A- C) Size distribution of 18- to 28-nt SINV-derived small RNAs of both strands (A), of only genomic or antigenomic strand (B), and the canonical duplexes with 3′ 2-nt overhangs of 22-nt vsiRNAs (C) sequenced from BHK cells infected with SINV at 24 hpi. Reads are shown as per million mature miRNAs. 5′ terminal nucleotide of vsRNAs is indicated by colour. 1U % of 21- to 23-nt vsiRNA is given in parenthesis. The percentage of 22-nt SINV vsRNAs with 2-nt 3′ overhangs in the total 22-nt vsRNAs was shown in box. (D, E) Size distribution, canonical duplexes with 3′ 2-nt overhangs of SINV-derived small RNAs sequenced from SINV-infected BALB/c suckling mice by i.p. (D) and C57BL/6 adult mice by intracranial injection (E). (F) Size distribution of total sequenced vsRNAs in the libraries constructed from Brainstem, Cerebellum, Striatum, Hippocampus and Cerebral Cortex of SINV-infected BALB/c adult mice (from left to right).

To investigate the virus-derived small RNAs in mammalian somatic cells, we analysed small RNA libraries from BHK-21 cells infected with SINV at 0.01 MOI. At twenty-four hours post-infection, we detected highly abundant total vsRNAs, but with no significant enrichment at 21-23nt (Figure 4(A)). The majority of the reads (98.6%) originated from the genomic strand of the virus and exhibited no defined peak at a specific length (Figure 4(B left)). Interestingly, the length distribution of the antigenomic small RNA reads peaked at the size of 22 nt (Figure 4(B right)). These vsRNAs were enriched for duplexes with a 20-nt perfect base-pairing and 2-nt 3′ overhangs (Figure 4(C)), indicating Dicer-dependent processing of the viral dsRNAs into viral siRNA duplexes.

We next sequenced the small RNAs from SINV infected WT suckling mice by intraperitoneal inoculation and WT adult mice by intracranial inoculation, respectively. In both profiles, we detected the canonical pairs of vsiRNAs in brain tissues, with the 22-nt vsiRNAs as the most abundant population and the 20-nt perfectly base-paired duplexes with 2-nt 3′ overhangs (Figure 4(D,E)). The SINV vsRNAs were distributed along the whole genome and enriched in the subgenomic RNA (Figure S6). Since NPCs mainly reside in the adult mammalian hippocampus of the brain and contribute to brain plasticity throughout life, these SINV vsiRNAs may also be generated from infected NPCs of the brain as same as ZIKV infection[11,43]. However, whether fully differentiated cells such as neuron and glial cells could generate vsiRNAs remains unknown. To address this question, we dissected the SINV infected mouse brain into five components and found uneven accumulation levels of SINV RNAs (Figure S7). Strikingly, we detected canonical SINV vsiRNAs not only from the hippocampus but also from the striatum and cerebral cortex, the two components with SINV RNA accumulation levels comparable to the hippocampus (Figure 4(F) and Figure S8). We next analyzed the expression levels of RNAi components in the five parts but detected no significant difference (Figure S9). This result indicated that the antiviral RNAi activated in CNS are able to produce abundant vsiRNAs upon SINV infections.

## Discussion

Recent studies have begun to provide evidence for the induction and suppression of a conserved antiviral RNAi response in mammal [2–4]. Production of abundant virus-derived small interfering RNAs (vsiRNAs) in mature mammalian cells or mice has been demonstrated for infection by mutant viruses defective in the expression of viral suppressor of RNAi [12–15]. However, there is still intense debate about whether this occurs under natural conditions because the small RNA sequencing from mammalian somatic cells or *in vivo* tissues infected by diverse wildtype viruses in failed to detect a dominant population of canonical vsiRNAs [2–4,44]. Recently, deep sequencing of mESCs infected with EMCV and hNPCs infected with ZIKV both revealed the accumulation of canonical vsiRNAs, but still didn't detect abundant vsiRNA generation in somatic cells [11,13]. Therefore, these studies argue for limited Dicer mediated generation of vsiRNAs in somatic cells [2,26]. Here, our results demonstrate that the vsiRNAs are induced by infection with at least two different types of wildtype viruses *in vivo*.

The use of mice with diminished or absent IFN-α/β signalling provides a small animal model for evaluating vaccines and therapeutics to combat ZIKV [28]. Such models have the advantage in studying the pathogenesis of ZIKV and mechanisms of viral immune evasion, and for understanding unexpected clinical manifestations of ZIKV infection in humans [16,27]. Although ZIKV replicates in a wide range of *Ifnar1*^−/−^ mice tissues, and viral RNAs can be detected in the serum during the course of infection, most of the previous studies on ZIKV only focus on the murine central nervous system and spinal cord for ZIKV causes neurodevelopmental defects in human fetuses [16,28]. In this work, we detected abundant viral RNAs and proteins in mice muscle tissue with ZIKV infection, demonstrating that this virus infects muscle tissue efficiently. We identified abundant somatic vsiRNAs generated from murine muscle tissues as well as central neuron vsiRNAs from the brain. We found that most of the vsiRNAs targeting ZIKV genome or antigenome were processed from the two terminal regions of the viral genomic RNAs (Figure 2(G,H)). This is different from what is found in mosquitoes and *ex vivo* hNPCs, which vsiRNAs cover the whole length of the ZIKV RNA and are equally mapped to the viral genome and antigenome [11,45]. These differences suggest that mammalian antiviral RNAi mechanisms *in vivo* have their own unique features distinct from the insects or *ex vivo* hNPCs. Moreover, these vsiRNAs of ZIKV can trigger homology-dependent viral RNA degradation in *Ifnar1*^−/−^ mice, suggesting the use of mice with diminished or absent IFN-α/β signalling a prospective animal model for studying antiviral RNAi. Our result reveals the importance to consider the effects of antiviral RNAi in this valuable mice model for future work.

SINV is one of the best-studied viruses extensively used in many applications, including the study of antiviral RNAi pathway in insects [46,47]. Many attempts have been unsuccessful in detecting canonical vsiRNAs in mammalian cell infection systems by SINV [48–50]. Our results suggested that the reason for the previous failure may be due to infection mode with SINV. Pfeffer and colleagues reported that the cellular antiviral endoribonuclease RNase L cleaves the SINV viral genome in HEK293 cells, producing small RNAs with no defined peak at a specific length and strong genomic strand bias [48]. Interestingly, they also showed a very small percentage of SINV small RNAs derived from the antigenomic strand exhibiting a size distribution with a peak at 22 nt [48]. This sub-profile of viral small RNAs is very similar to what we observed in BHK cells with SINV infection and Vero cells with ZIKV infection. Our results also explain the previous study that Dicer ^−/−^ cells are more permissive to SINV replication *in vitro* than wild type cells [51]. In line with the aforementioned studies, the activity of RNase L is easily triggered by virus infection in cell culture and produces abundant viral small RNAs of no defined peak at a specific length which would mask the canonical vsiRNAs in the infection experiments. But *in vivo*, the activity of RNase L is highly regulated because excessive activities can adversely affect the host [52]. Such differences may be the reason why we can detect canonical vsiRNAs from the mouse brain tissue rather than cell culture systems with SINV infections.

Virus clearance by the immune system is a major challenge when RNA viruses infect CNS because any inflammation to CNS can be damaging or even fatal [39,40,42]. It's indicated that CNS requires noncytolytic, rather than cytolytic, immune mechanisms for virus clearance [34,41]. Therefore, as a small RNA-based immune response by noncytolytic viral clearance, the antiviral RNAi pathway in CNS is worthy of further study [1]. Our results for the first time indicate the “hallmark” of vsiRNAs are generated from multiple components of mice brain with SINV infection. This indicates the possibility that antiviral RNAi is part of the antiviral immune strategy in the CNS upon virus infection, and the immune responses in the CNS are highly regulated.

## Supplementary Material

Supplemental Material
