# Supplementary material for: The activation of antiviral RNA interference not only exists in neural progenitor cells but also in somatic cells in mammals
Source: Emerg Microbes Infect. 2020 Jul 9;9(1):1580–9. doi: 10.1080/22221751.2020.1787798 (PMC7473182; doi:10.1080/22221751.2020.1787798)
Supplement: Supplemental Material [file TEMI_A_1787798_SM1076.docx]

# SUPPLEMENTAL INFORMATION

## Supplemental Figures (1-9)


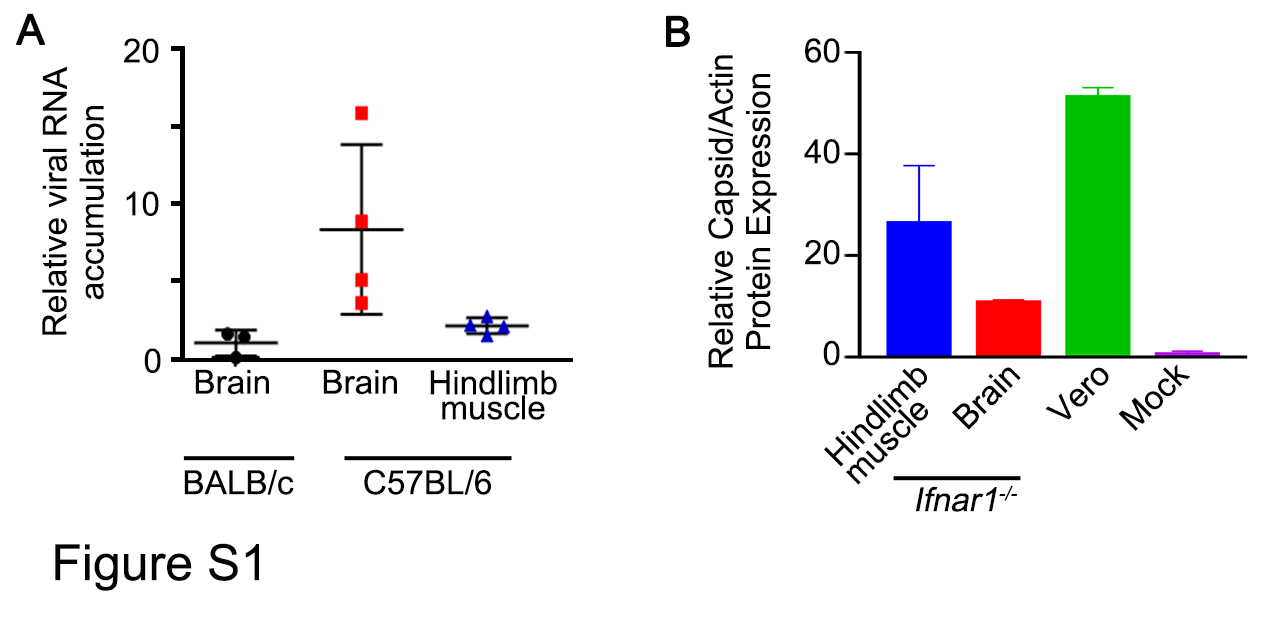


Figure S1. (A) The relative viral accumulation determined by RT-qPCR from brain and hindlimb muscle of BALB/c and C57BL/6 suckling mice infected with ZIKV (n=3~4 per group). (B) The relative ZIKV capsid protein expression of Figure 2B normalized to that of actin was quantified with Image J software.


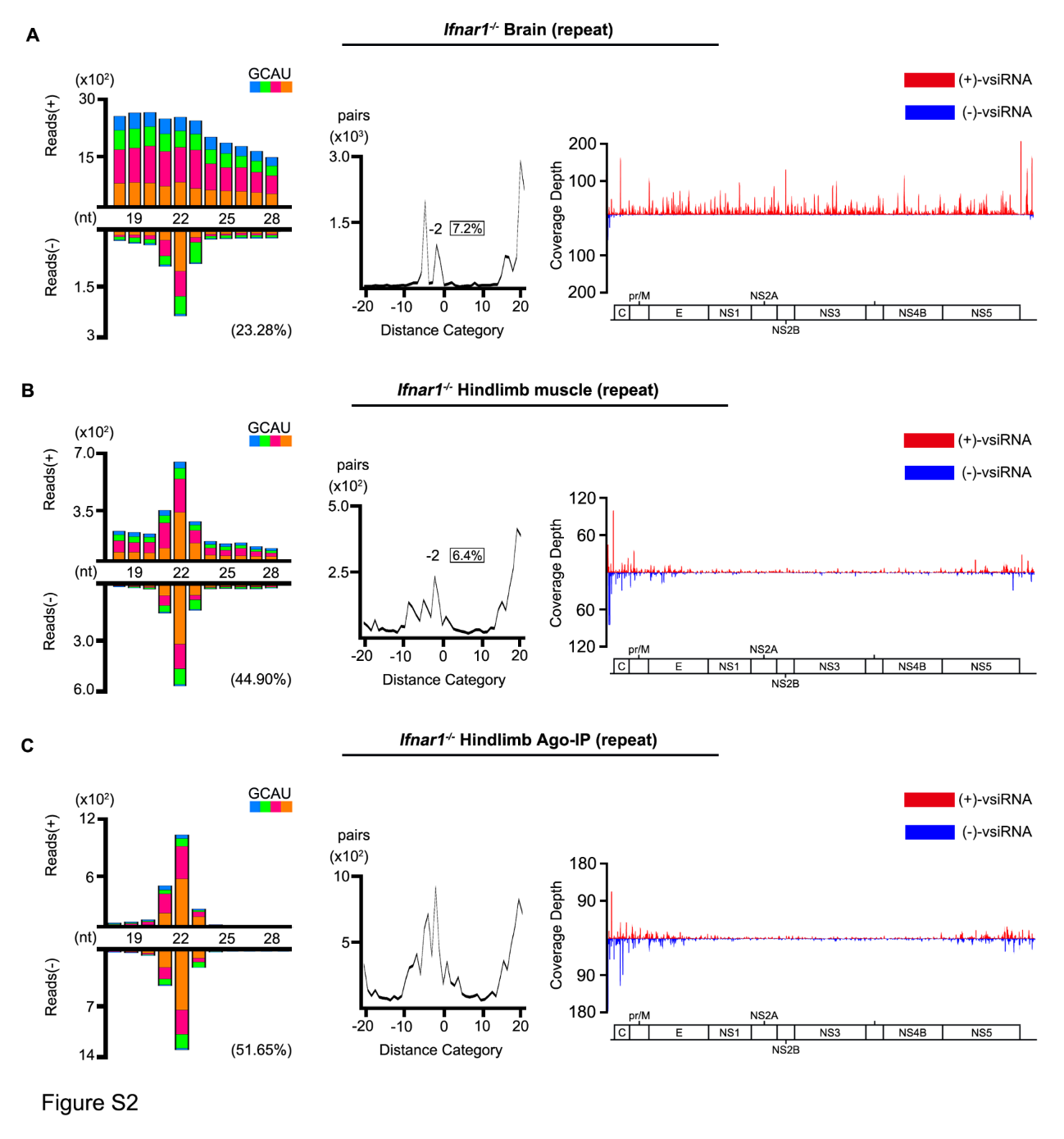


Figure S2. Size distribution, canonical duplexes with 3’ 2-nt overhangs of virus derived small RNAs and viral genomic coverage depth of each nucleotide position by 21- to 23-nt vsiRNAs sequenced from brain (A) and hindlimb muscle (B) of *Ifnar1^-/-^* suckling mice infected with ZIKV at 4 dpi, either without (A, B) or with (C) co-immunoprecipitation by antibodies specific to AGOs (a repeat of Figure 2(C-E)). Reads are shown as per million mature miRNAs. 5’ terminal nucleotide of vsRNAs is indicated by color. 1U% of 21- to 23-nt vsiRNA in each library is shown in parentheses. The “-2” peak corresponded to the pair of canonical 22-nt vsiRNAs with a 20-nt duplex region plus 2-nt 3’ overhangs, calculated by an algorithm described previously counting pairs of complementary 22-nt vsiRNAs in each distance category (in nucleotides) between 5’ and 3’ ends of each pair. Length (nt) indicates x-axis for size distribution. Distance category indicates x-axis for duplex pattern. The percentage of 22-nt SINV vsRNAs with 2-nt 3’ overhangs in the total 22-nt vsRNAs was shown in box.


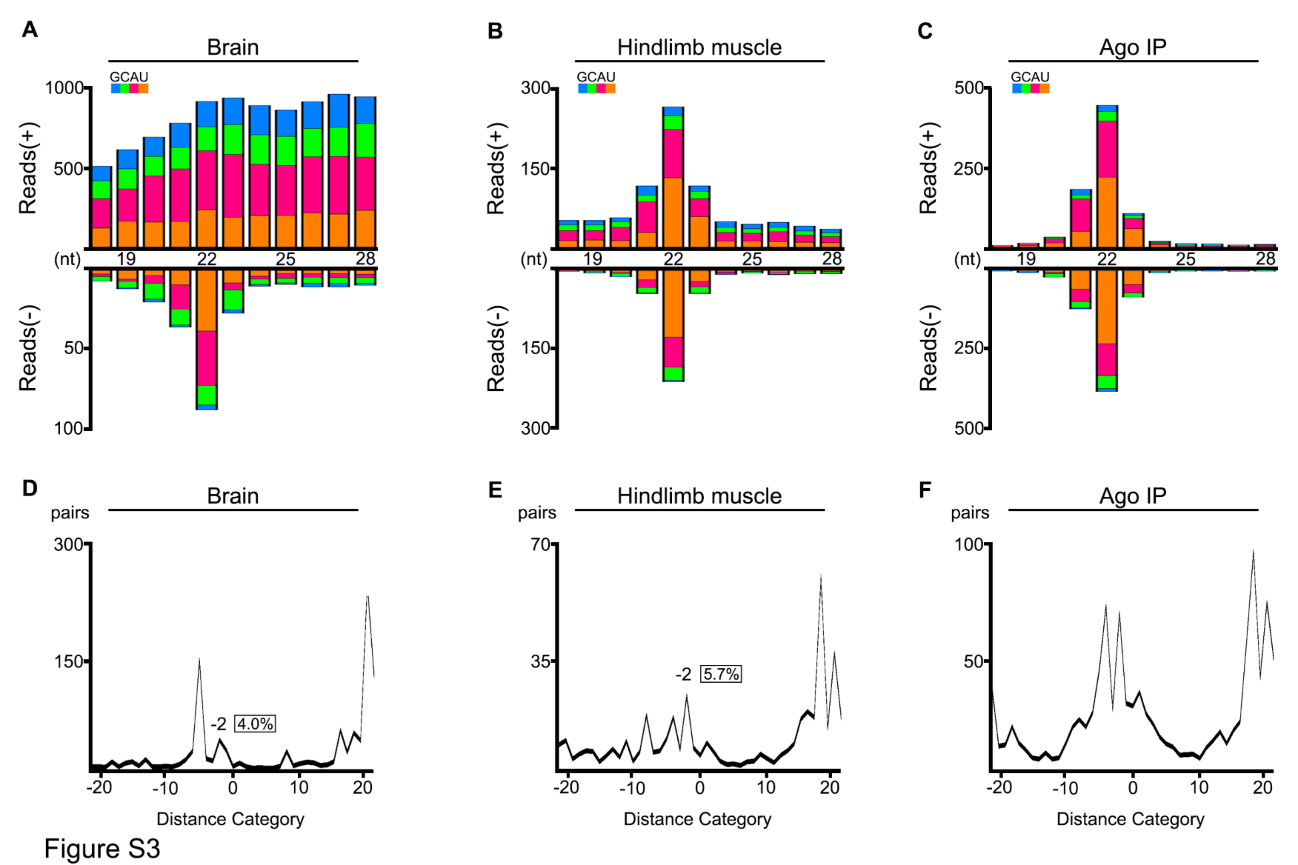


Figure S3. Size distribution of vsiRNAs normalized by per million total reads (A-C) and canonical duplexes with 3’ 2-nt overhangs of virus derived small RNAs normalized by per million mature miRNAs (D-F) sequenced for Figure 2C-E, from brain (A, D) and hindlimb muscle (B, C, E, F) of *Ifnar1^-/-^* suckling mice infected with ZIKV at 4 dpi, either without (A, B, D, E) or with (C, F) co-immunoprecipitation by antibodies specific to AGOs. The percentage of 22-nt SINV vsRNAs with 2-nt 3’ overhangs in the total 22-nt vsRNAs was shown in box.


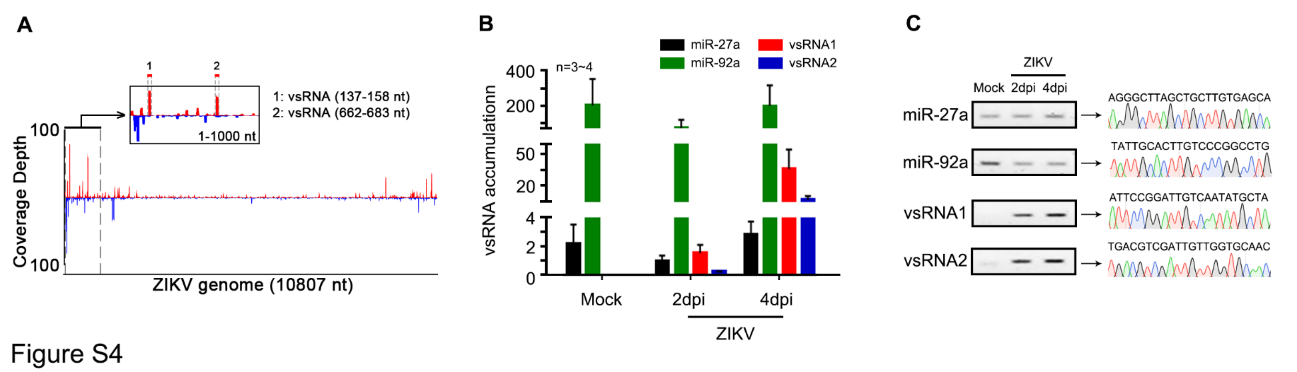


Figure S4. ZIKV-derived vsiRNAs detected by stem-loop RT-qPCR. (A) The profile of 1-1000nt viral genomic coverage depth of each nucleotide position by 21- to 23-nt vsiRNAs sequenced from hindlimb muscle of *Ifnar1^-/-^*suckling mice infected with ZIKV at 4 dpi and vsiRNAs detected by stem-loop RT-qPCR were highlighted in the diagram. (B) The relative abundance of ZIKV vsiRNAs and two miRNAs in *Ifnar1^-/-^* suckling mice challenged with culture medium DMEM or ZIKV at 2 and 4dpi were detected by stem-loop RT-qPCR. (C) The products of stem-loop RT-qPCR were analysis by agarose gel electrophoresis and confirmed by Sanger.


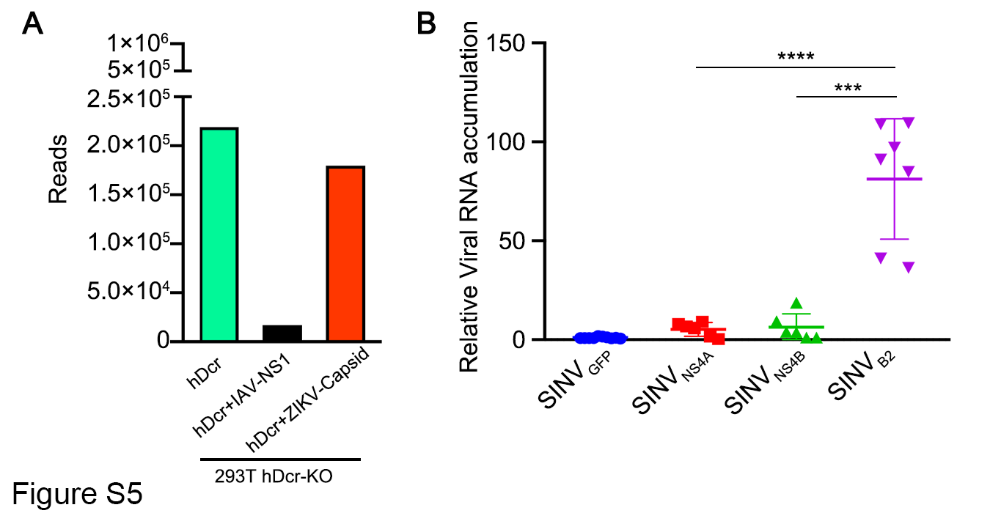


Figure S5. (A) Counts (per million 18- to 28-nt total reads) of IAV-derived siRNAs sequenced from the cells in Figure 3D infected by PR8/delNS1 (A repeat of Figure 3E). (B) The relative viral accumulation levels determined by RT-qPCR from hindlimb muscle of BALB/c suckling mice infected with SINV_GFP_, SINV_NS4A_, SINV_NS4B_, SINV_B2_ at 3dpi (n=6 per group). *** indicates p<0.001, **** indicates p<0.0001, Student’s t-test.


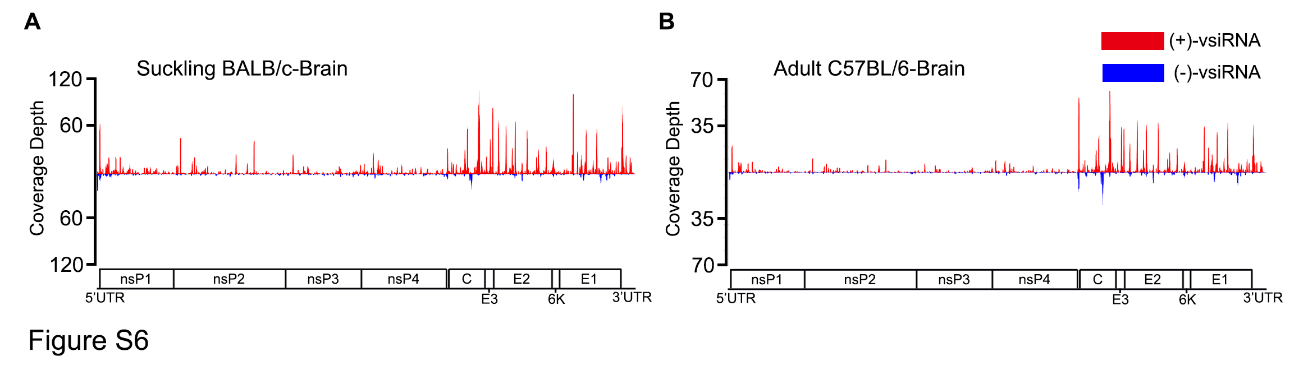


Figure S6. Genomic coverage depth by 21- to 23-nt vsiRNAs sequenced from SINV-infected BALB/c suckling mice by i.p. (A) and C57BL/6 adult mice by intracranial injection (B).


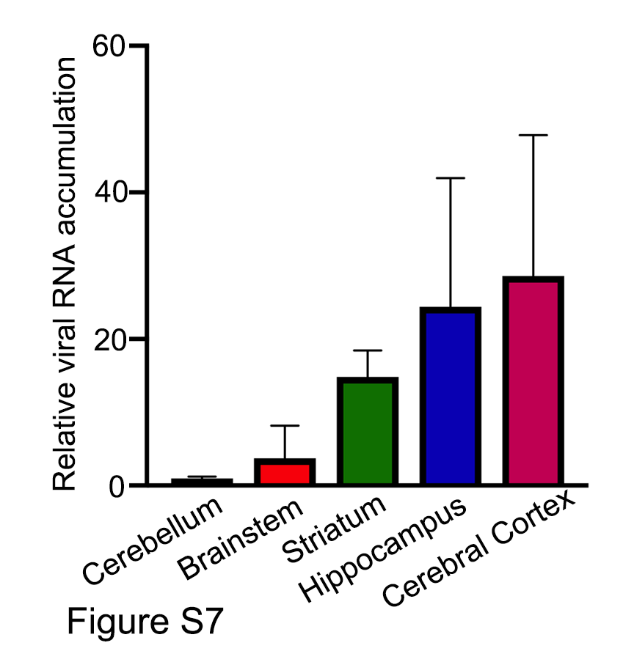


Figure S7. The relative viral RNA levels of SINV determined by quantitative RT-qPCR in five different brain components of the BALB/c adult mice (n=3 per group). Primers were shown in Table S1.


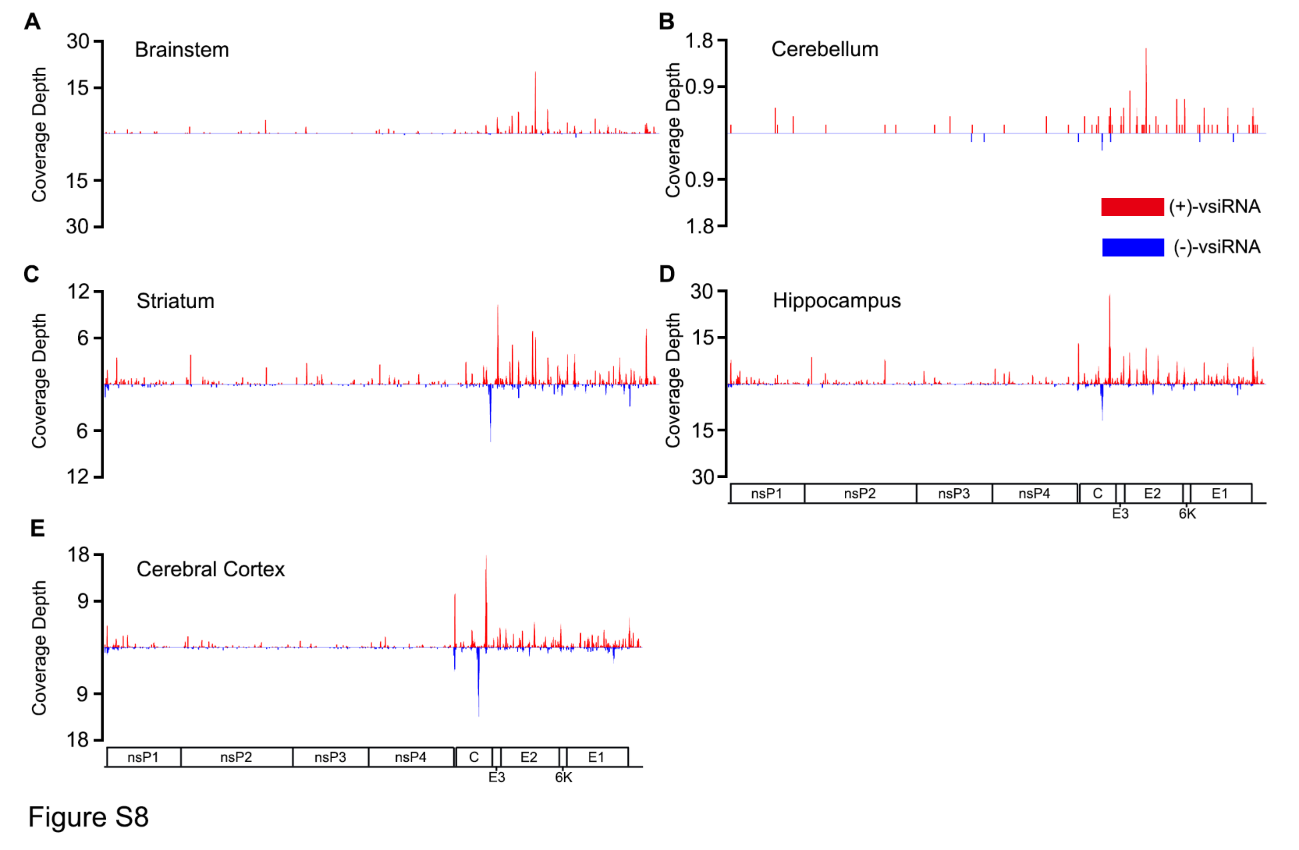


Figure S8. Genomic coverage depth by 21- to 23-nt vsiRNAs sequenced from Brainstem, Cerebellum, Striatum, Hippocampus and Cerebral Cortex of SINV-infected BALB/c adult mice by intracranial injection.


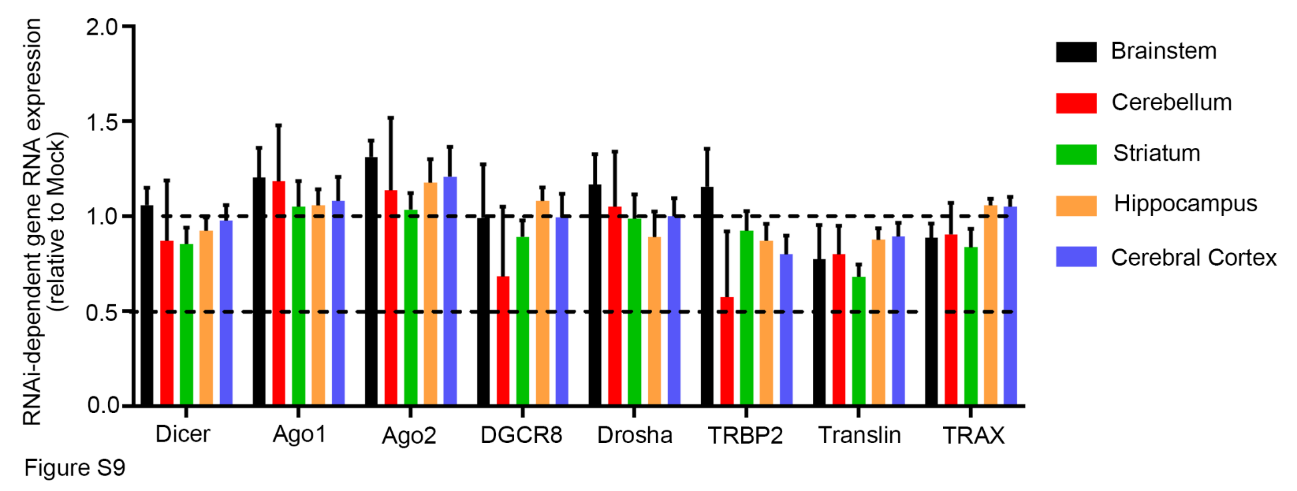


Figure S9. Differential expression of RNAi pathway related genes in five different brain components of the BALB/c adult mice with SINV infection (n=3 per group).

**Supplemental Tables S1-3**

Table S1: Primers, Related to Experimental Procedures

| Name | Sequence |
| --- | --- |
|  |  |
| Actin-Mice mRNA-forward | 5’ATTGGCAACGAGCGGTTCC3’ |
| Actin-Mice mRNA-reverse | 5’AGCACTGTGTTGGCATAGAGG3’ |
| ZIKV NS5 mRNA-forward | 5’CCTTGGATTCTTGAACGAGGA3’ |
| ZIKV NS5 mRNA-reverse | 5’AGAGCTTCATTCTCCAGATCAA3’ |
| SINV nsP2 mRNA-forward | 5’GGCGTTTCGCACTAAGAAAG3’ |
| SINV nsp2 mRNA-reverse | 5’TGCAGCAGTTTTTCCTCCTT3’ |
| Dicer mRNA-forward | 5’GACCGTGTTCCTCGTCAACTCTG3’ |
| Dicer mRNA-reverse | 5’TCAAAACAGTCAAGGCGACATAGC3’ |
| Ago-1 mRNA-forward | 5’TCGGAAGATTTCCAAGGATG3’ |
| Ago-1 mRNA-reverse | 5’GTTGCCATTCCCAAGAGTGT3’ |
| Ago-2 mRNA-forward | 5’AAGTCGGACAGGAGCAGAAA3’ |
| Ago-2 mRNA-reverse | 5’GAAACTTGCACTTCGCATCA3’ |
| DGCR8 mRNA-forward | 5’GCTGCAGGAGTAAGGACAGG3’ |
| DGCR8 mRNA-reverse | 5’TCGAGCACTGCATACTCCAC3’ |
| Drosha mRNA-forward | 5’GGACCATCACGAAGGACACT3’ |
| Drosha mRNA-reverse | 5’GATGTACAGCGCTGCGATAA3’ |
| TRBP2 mRNA-forward | 5’CTTCCAAAAAGCTGGCAAAG3’ |
| TRBP2 mRNA-reverse | 5’GAGCAACTGCGAAGGGATAG3’ |
| Translin mRNA-forward | 5’TACTTCAAGGGGTCCACCAG3’ |
| Translin mRNA-reverse | 5’TCACCAGGGTCTCTGTTTCC3’ |
| TRAX mRNA-forward | 5’GATGCTGGCCTTCAAATCAT3’ |
| TRAX mRNA-reverse | 5’GACAGCTCTTGGGCTACCTG3’ |

Table S2: Primers for stem-loop qPCR

| Name | Sequence |
| --- | --- |
|  |  |
| Stem-loop primer for miR-27a-5p | 5’GTCGTATCCAGTGCAGGGTCCGAGGTATTCGCACTGGATACGACTGCTCA3’ |
| Primer for miR-27a-5p qPCR-forward | 5’AGGGCTTAGCTGCTTG3’ |
| Stem-loop primer for miR-92a-3p | 5’GTCGTATCCAGTGCAGGGTCCGAGGTATTCGCACTGGATACGACCAGGCC3 |
| Primer for miR-92a-3p qPCR-forward | 5’TATTGCACTTGTCCC3’ |
| Stem-loop primer for vsRNA1 | 5’GTCGTATCCAGTGCAGGGTCCGAGGTATTCGCACTGGATACGACTAGCAT3’ |
| Primer for vsRNA1 qPCR-forward | 5’ATTCCGGATTGTCAAT3’ |
| Stem-loop primer for vsRNA2 | 5’GTCGTATCCAGTGCAGGGTCCGAGGTATTCGCACTGGATACGACGTTGCA3’ |
| Primer for vsRNA2 qPCR-forward | 5’TGACGTCGATTGTTGG3’ |
| Primer for qPCR-reverse | 5’GTGCAGGGTCCGAGGT3’ |

Table S3: Contents and properties of the small RNA libraries sequenced

| Library | Total reads  (18-28nt) | miRNA^1^  (mature) | Virus reads  (18-28nt) | Virus reads of 21- to 23-nt | | | |
| --- | --- | --- | --- | --- | --- | --- | --- |
|  |  |  |  | Reads | % of miRNA | % of all sizes | (+) strand % |
| ZIKV: Vero 5dpi. | 17,066,927 | 2,034,134^2^ | 412,070 | 120,176 | 5.9% | 29.2% | 99.9% |
| ZIKV: BALB/c suckling mice 8dpi. Brain | 28,464,004 | 10,029,166 | 4,198 | 1,342 | 0.01% | 32.0% | 94.9% |
| ZIKV: C57BL/6 suckling mice 5dpi. Brain | 28,325,737 | 10,482,988 | 139 | 45 | 0.0004% | 32.4% | 97.8% |
| ZIKV: C57BL/6 suckling mice 5dpi. Hindlimb | 29,498,294 | 15,691,903 | 221 | 123 | 0.001% | 55.7% | 65.0% |
| ZIKV: *Ifnar1*^-/-^ suckling mice 4dpi. Brain | 15,070,382 | 4,786,927 | 139,367 | 41,931 | 0.9% | 30.1% | 94.6% |
| ZIKV: *Ifnar1*^-/-^ suckling mice 4dpi. Hindlimb | 23,083,324 | 10,853,497 | 27,894 | 18,314 | 0.2% | 65.7% | 62.2% |
| ZIKV: *Ifnar1*^-/-^ suckling mice 4dpi. Ago IP | 12,128,980 | 5,750,105 | 17,815 | 16,018 | 0.3% | 89.9% | 55.4% |
| ZIKV: *Ifnar1*^-/-^ suckling mice 4dpi. Brain (Repeat) | 22,760,078 | 9,246,712 | 220,309 | 71,373 | 0.8% | 32.4% | 94.5% |
| ZIKV: *Ifnar1*^-/-^ suckling mice 4dpi. Hindlimb (Repeat) | 18,751,682 | 10,810,738 | 35,253 | 22,658 | 0.2% | 64.3% | 58.4% |
| ZIKV: *Ifnar1*^-/-^ suckling mice 4dpi. Ago IP (Repeat) | 25,822,619 | 13,719,665 | 55,583 | 50,354 | 0.4% | 90.6% | 46.6% |
| SINV: BHK 24hpi | 16,272,505 | 266,137 | 911,171 | 205,623 | 77.3% | 22.6% | 98.6% |
| SINV: BALB/c suckling mice 3dpi. Brain mix | 19,557,841 | 6,432,885 | 56,805 | 21,708 | 0.3% | 38.2% | 87.4% |
| SINV: C57BL/6 adult mice 3dpi. Brain | 24,795,558 | 7,827,552 | 28,708 | 11,954 | 0.2% | 41.6% | 84.2% |
| SINV: BALB/c adult mice 3dpi. Brainsterm | 19,755,085 | 4,596,200 | 2,110 | 634 | 0.01% | 30.0% | 97.3% |
| SINV: BALB/c adult mice 3dpi. Cerebellum | 19,166,466 | 6,004,340 | 342 | 98 | 0.002% | 28.7% | 91.8% |
| SINV: BALB/c adult mice 3dpi. Striatum | 33,013,570 | 10,166,147 | 5,293 | 2,166 | 0.02% | 40.9% | 75.7% |
| SINV: BALB/c adult mice 3dpi. Hippocampus | 17,246,278 | 5,712,029 | 6,988 | 2,811 | 0.05% | 40.2% | 82.1% |
| SINV: BALB/c adult mice 3dpi.Cerebral Cortex | 20,207,809 | 7,058,829 | 4,228 | 1,933 | 0.03% | 45.7% | 72.6% |
| PR8ΔNS1: 293T-Dicer KO +hDicer | 9,108,277 | 207,754 | 768,765 | 684,571 | 329.5% | 89.0% | 32.4% |
| PR8ΔNS1: 293T-Dicer KO +hDicer +ZIKV Capsid | 10,447,553 | 240,666 | 491,980 | 437,349 | 181.7% | 88.9% | 32.5% |
| PR8ΔNS1: 293T-Dicer KO +hDicer +IAV NS1 | 13,141,437 | 318,044 | 45,121 | 26,111 | 8.2% | 57.9% | 30.9% |
| PR8ΔNS1: 293T-Dicer KO +hDicer (Repeat) | 7,596,074 | 257,392 | 1,665,397 | 1,478,077 | 574.3% | 88.8% | 44.6% |
| PR8ΔNS1: 293T-Dicer KO +hDicer +ZIKV Capsid (Repeat) | 9,704,636 | 335,484 | 1,745,040 | 1,549,900 | 462.0% | 88.8% | 44.2% |
| PR8ΔNS1: 293T-Dicer KO +hDicer +IAV NS1 (Repeat) | 8,052,400 | 301,230 | 135,689 | 95,564 | 31.7% | 70.4% | 40.6% |

1 Indicating the reads perfectly identical to mature microRNAs

2 Reference to mice miRNA for Vero miRNA data not available
